# Nutritional Risk Assessment of Patients Undergoing Pancreaticoduodenectomy After Standardization of Preoperative Nutritional Support

**DOI:** 10.3390/nu17172871

**Published:** 2025-09-04

**Authors:** Katerina Knapkova, Martin Lovecek, Jana Tesarikova, Michal Gregorik, Stefan Kolcun, Dusan Klos, Pavel Skalicky

**Affiliations:** 1 Department of Surgery, Faculty of Medicine and Dentistry, Palacky University Olomouc, Zdravotníků 248/7, 77900 Olomouc, Czech Republic; katerina.knapkova@fnol.cz (K.K.); jana.tesarikova@fnol.cz (J.T.); dusan.klos@fnol.cz (D.K.); 2 Department of Surgery, University Hospital Olomouc, Zdravotníků 248/7, 77900 Olomouc, Czech Republic; martin.lovecek@fnol.cz (M.L.); michal.gregorik@fnol.cz (M.G.); stefan.kolcun@fnol.cz (S.K.)

**Keywords:** nutritional risk, morbidity, preoperative nutritional support

## Abstract

**Background/Objectives**: Nutritional status affects postoperative outcomes, but the effect of standardized preoperative nutritional preparation on morbidity in malnourished patients undergoing pancreatoduodenectomy (PD) remains unclear. This study evaluated preoperative nutritional parameters following the standardization of nutritional screening and intervention in patients undergoing PD. The influence of nutritional parameters on postoperative morbidity was also assessed. **Methods**: This prospective cohort study was conducted from 2019 to 2021 at the Department of Surgery, University Hospital, Olomouc. A total of 133 patients were categorized nutritionally as “high risk” (weight loss or reduced appetite with restricted intake) or “low risk” (no weight or appetite loss). High-risk patients received enteral supplementation of 600 kcal/day. A multivariate logistic regression model was used to evaluate the association between major postoperative complications and risk factors, including sex, age, ASA score, BMI, weight and appetite loss, malignancy, duct diameter, pancreatic texture, serum albumin, prealbumin, MUST, and NRS2002 scores. **Results**: Eighty patients (60.2%) were “high risk,” and 53 (39.8%) were “low risk.” Major morbidity and 90-day mortality occurred in 24 (18.0%) and 4 (3.0%) patients, respectively. No significant differences were observed between high- and low-risk groups in CD morbidity grade, 90-day mortality, POPF, PPH, DGE, or hospital stay. Major morbidity was associated with prealbumin < 0.2 g/L, duct diameter ≤ 3 mm, soft texture, and male sex, with respective odds ratios of 3.307, 3.288, 4.814, and 2.374. **Conclusions**: High-risk patients receiving preoperative nutrition had comparable rates of major complications and POPF as low-risk patients. Low serum prealbumin predicts major postoperative complications after PD.

## 1. Introduction

Extensive surgical interventions trigger substantial metabolic and nutritional alterations, primarily driven by the activation of inflammatory pathways and secretion of stress-related hormones and cytokines. The magnitude of this physiological reaction tends to correlate with the severity of the surgical insult [1]. Among abdominal surgeries, pancreaticoduodenectomy (PD) is among the most complex procedures, owing to its extensive scope of resection, systemic physiological burden, and elevated rate of postoperative complications [2,3]. Effective tissue repair depends on a well-regulated metabolic response, which requires an adequate supply of both macro-and micronutrients. Nutritional stores can be quickly exhausted in malnourished individuals or those who experience major postoperative complications, potentially hindering healing.

Many patient-specific factors in individuals referred for PD contribute to the high rate of preoperative malnutrition in this population. These include exocrine pancreatic insufficiency caused by reduced enzyme production due to diminished pancreatic parenchyma or pancreatic duct obstruction. Nausea and dyspepsia are often associated with duodenal disorders, impaired gastric evacuation, and biliary obstruction. In patients with pancreatic cancer, chronic subclinical inflammation and metabolic demands of the tumor contribute to malnutrition [4].

The most common complications following PD are pancreatic fistula (POPF), post-pancreatectomy hemorrhage (PPH), and delayed gastric emptying (DGE) [5,6,7]. The main well-documented risk factors for increased morbidity include soft pancreatic texture, a pancreatic duct diameter < 3 mm, and male sex [8]. All of these factors are virtually uncontrollable preoperatively and are related to the characteristics of the individual patient and the type of disease. In contrast, nutritional status may be influenced by preoperative nutritional preparation. According to a single study, preoperative nutritional support was a significant protective factor against POPF in patients at high nutritional risk (OR = 0.339) [9]. With an average of 622 pancreatic resections performed annually in the Czech Republic during the last monitored period [10], a reduction in complications could significantly reduce healthcare costs and benefit patients.

The recommendations of the European Society for Nutrition and Metabolism (ESPEN) include nutritional intervention in patients with identified nutritional risks before planned major surgery [11]. Severe malnutrition risk is determined using the Subjective Global Assessment (SGA) Grade C or a Nutritional Risk Screening 2002 (NRS2002) score > 5. Another group recommended to receive oral nutritional supplements regardless of their formal nutritional status are patients who are unable to meet their energy needs from their normal food intake, all malnourished patients with cancer, and patients at high risk undergoing major abdominal surgery. In these patients, nutritional therapy should be initiated immediately after screening. Evidence on the positive impact of nutritional preparations in reducing complications in pancreatic surgery is limited [9,12]. Furthermore, the use of the NRS2002 did not correlate with the incidence of postoperative complications, and its clinical utility remains controversial [13].

Given the limited and inconclusive data on the efficacy of standardized nutritional support in this high-risk population, the primary objective of this study was to evaluate preoperative nutritional parameters in patients undergoing PD after the standardization of nutritional screening and interventions. A secondary objective was to evaluate the impact of preoperative nutritional status on postoperative morbidity and mortality.

To our knowledge, this is the first prospective study from Central Europe to systematically implement and evaluate a standardized preoperative nutritional support protocol in an unselected cohort of patients undergoing PD. The novelty of our work lies in comparing outcomes between nutritionally stratified groups based on pragmatic clinical criteria, thereby assessing the real-world effectiveness of this intervention in mitigating malnutrition-related surgical risk.

## 2. Materials and Methods

### 2.1. Study Design and Population

A prospective, single-center cohort study with a standardized, non-randomized nutritional intervention targeted at a predefined high-risk group was conducted between 2019 and 2021 at the Department of Surgery of University Hospital Olomouc, a high-volume center in the Czech Republic. All consecutive patients who underwent PD for pancreatic, distal bile duct, or duodenal pathology between January 2019 and December 2021 were included in this study.

### 2.2. Sample Size and Sampling Procedure

Statistical power analysis was performed based on the estimated prevalence of POPF and major morbidity of 15%. Assuming a target sensitivity and specificity of 95% and a confidence interval of 0.05, the minimum required number of patients was calculated as 109.

### 2.3. Study Procedures

All patients underwent preoperative evaluation by a nutrition professional as part of the nutritional risk screening process at the time of surgical planning. Patients were classified into the “high-risk” malnutrition cohort if they met at least one of the following criteria: (a) any weight loss within the previous six months or (b) reduced appetite accompanied by restricted oral intake. The criteria of weight loss and reduced appetite were chosen as primary indicators of nutritional risk due to their high prevalence and clinical significance in patients with pancreatic and periampullary diseases, representing key components of cancer-associated cachexia. All patients in the “high-risk” cohort underwent nutritional intervention consisting of supplementation with a standard polymeric nutritionally complete oral supplement providing approximately 30 g of protein and 75 g of carbohydrates per 600 kcal daily dose. Nutritional intervention was initiated on the day of nutritional screening and terminated one day before surgery. The remaining patients were assigned to the malnutrition “low-risk” cohort and did not receive any targeted nutritional intervention. All PDs were performed using an open surgical technique, either in Whipple’s modification or pylorus-preserving PD in Traverso’s modification, with pancreatojejunal anastomosis performed according to Catell’s technique.

### 2.4. Data Collection

A prospectively maintained database was used to collect various clinical and perioperative variables, including sex, age, American Society of Anesthesiologists classification (ASA), pathology underlying the resection, pancreatic texture, pancreatic duct diameter, duration of surgery, perioperative blood loss, length of hospital stay, postoperative morbidity according to the Clavien-Dindo classification (CD) [14], and 30-day and 90-day mortality rates. Complications of Clavien-Dindo grades III–V were considered major postoperative complications. Specific complications of pancreatic resection (POPF, DGE, and PPH) were classified according to the International Study Group of Pancreatic Surgery [5,6,7]. Postoperative monitoring was performed daily during postoperative hospitalization and once a week during outpatient checkups for at least 90 days. Pancreatic texture was assessed intraoperatively by the primary surgeon through manual palpation of the pancreatic remnant. It was categorized dichotomously as ‘soft’ or ‘medium/hard’ based on the surgeon’s subjective assessment of gland firmness and fibrosis. Time to surgery was defined as the interval between the dates of nutritional screening and surgery. Clinical nutritional parameters, including preoperative BMI, total weight loss, and lack of appetite, were assessed at the time of nutritional screening. The preoperative serum albumin and prealbumin levels were measured on the day of admission for surgery.

### 2.5. Data Analysis

Categorical variables are presented as absolute numbers and percentages, whereas continuous variables are reported as medians with ranges (minimum–maximum). The normality of data distribution was assessed using the Shapiro–Wilk test. Both univariable and multivariable logistic regression analyses were performed to evaluate the relationship between risk factors and major postoperative complications. Odds ratios (ORs) with 95% confidence intervals and two-sided *p*-values < 0.05 were calculated. The univariable analysis was performed for the following variables: age, sex, ASA score, BMI, weight loss, lack of appetite, albumin, prealbumin, presence of malignancy, pancreatic texture, and pancreatic duct diameter. The multivariable model was subsequently constructed using only those variables that reached statistical significance in the univariable analysis. IBM SPSS Statistics version 23 was used for the statistical analysis.

### 2.6. Ethical Considerations

Ethical approval for the study was granted by the Institutional Ethics Committee of University Hospital Olomouc (Approval No. 159/16), and all participants provided written informed consent.

## 3. Results

### 3.1. Participant Selection

In total, 133 patients were enrolled in this study. A total of 80 (60.2%) patients met the criteria for inclusion in the “high risk” malnutrition cohort: 70 (52.6%) met the weight loss criterion, and 12 (9.0%) had a lack of appetite associated with oral intake restrictions (two patients met both criteria). The remaining 53 (39.8%) patients constituted the “low-risk” malnutrition cohort (see Table 1).

### 3.2. Demographic, Clinicopathological and Histopathological Characteristics

The cohort included 80 (60.2%) men and 53 (39.8%) women, with a median age of 64.2 years (range: 36–84 years). Most patients were classified as ASA II (*n* = 111/133; 84.1%), with fewer classified as ASA I (*n* = 2/133; 1.5%) or ASA III (*n* = 19/133; 14.4%). The mean BMI was 25.9 (17.5–38.6) kg/m^2^ (see Table 1 for details).

The largest group comprised patients referred for PD due to ductal adenocarcinoma of the pancreatic head (*n* = 74/133, 55.6%). A total of 37 (27.8%) patients had soft pancreatic texture, and the most common pancreatic duct diameter was less than 3 mm in 52 (39.4%) patients. Low albumin and low prealbumin levels were present in 7 (5.3%) and 14 (10.5%) patients, respectively. There were no significant differences between the two patient cohorts in sex, age, BMI, ASA score, histopathological diagnosis, pancreatic texture, pancreatic duct diameter, or albumin and prealbumin levels.

More than half of the patients reported weight loss at the time of screening (70/133; 52.6%), most commonly in the range of 5–10 kg in 50 patients (37.6%). A MUST score ≥ 2 and an NRS2002 score ≥ 3 were recorded in 12 (9.0%) and 72 (54.1%) patients, respectively, all of these patients belonged to the ‘high-risk’ cohort. The demographic and clinicopathological data of the patients are presented in Table 1.

### 3.3. Operative and Postoperative Characteristics

The operative and postoperative data are presented in Table 2. Of the 133 PD performed, 121 (91.0%) were pylorus-preserving resections and 12 (9.0%) were Whipple procedures. The incidence of complications in the overall patient population was CD grade II in 73 (54.9%) and CD grades III–V in 24 (18.0%) patients. The 90-day mortality occurred in 4 (3.0%) patients, and the average length of hospital stay was 14 days (range, 7–128 days). POPF types B and C occurred in 16 (12.0%) and 7 (5.3%) patients, respectively, whereas PPH types A, B, and C occurred in zero (0.0%), seven (5.3%), and 5 (3.8%) patients. The median time to surgery was 21 days (range: 5–42 days). There were no significant differences between the “high-risk” and “low-risk” malnutrition cohorts in CD morbidity grade, 30-day and 90-day mortality, POPF, PPH, DGE, time to surgery, operative time, hospital stay, or blood loss.

### 3.4. Risk Factors for Major Postoperative Morbidity

The findings of the univariate analysis assessing the relationship between major postoperative morbidity and selected clinical variables are summarized in Table 3. In multivariate analysis, major postoperative morbidity was significantly associated with a preoperative prealbumin level < 0.2 g/L (OR: 3.815; 95% CI: 1.081–13.464, *p* = 0.037), pancreatic duct width ≤ 3 mm (OR: 3.507; 95% CI: 1.336–9.206, *p* = 0.011), soft pancreatic texture (OR: 4.331; 95% CI: 1.828–10.261, *p* = <0.001) and male sex (OR: 2.240; 95% CI: 1.096–4.578, *p* = 0.027) (see Table 3). Other potential risk factors—including age, ASA score, BMI < 18.5, lack of appetite, weight loss, presence of malignancy, albumin level, MUST, and NRS2002—had no statistically significant association with major postoperative morbidity.

## 4. Discussion

One of the objectives of this study was to analyze the preoperative nutritional parameters of patients undergoing PD and to quantify malnutrition in this patient cohort. As our data show, the median BMI of patients in the “high-risk” malnutrition cohort was 25.8 kg/m^2^, which falls within the WHO-defined overweight category [15]. Relying on this parameter alone is therefore not a suitable classification criterion for assessing the risk of patients undergoing PD. Patients with pancreatic cancer have one of the highest prevalence rates of malnutrition and cachexia among all cancer diagnoses. Unintentional weight loss is often the first and most prominent symptom, affecting up to 80% of patients at the time of diagnosis [16]. Loss of appetite is another key and early symptom. These two criteria are therefore highly sensitive and clinically pragmatic indicators for immediate risk identification in this specific cohort and were also used in the patient classification methodology in our study.

The median time between nutritional screening and surgery in our cohort was 21 days, which was primarily determined by surgical scheduling. Perioperative malnutrition has been identified as a major modifiable risk factor for postoperative morbidity and mortality in the National Surgical Quality Improvement Program (NSQIP) [17]. Several studies on prehabilitation before pancreatic surgery suggest benefits on postoperative outcomes; however, standardized protocols regarding the form, composition, and duration of nutritional preparation in PD are lacking [18,19]. All patients in the malnutrition “high-risk” cohort were prescribed 600 kcal/day of enteral supplementation, based on the general ESPEN recommendations for major surgery [11]. The optimal duration of preoperative preparation in patients undergoing PD remains controversial due to limited evidence in the current literature. Although extended nutritional preparation may improve the nutritional status of patients with malignancy, it carries the risk of cancer progression during the delay and may increase the number of patients requiring preoperative biliary drainage, which is associated with higher postoperative morbidity [20]. Our study did not include a predefined duration for preoperative preparation; therefore, definitive conclusions and recommendations regarding its optimal length cannot be drawn, and further prospective studies are warranted.

Our study showed that there was no difference in malnutrition risk among patients diagnosed with PDAC, other periampullary malignancies, or benign pancreatic diseases. This finding contradicts previously published results that reported worse nutritional conditions in patients with malignant diseases [21]. One possible explanation is the variation in criteria and timing for pancreatic resection in patients with chronic pancreatitis across different study periods [22]. Another reason may be that the operable tumor stages eligible for PD are typically not associated with severe tumor cachexia, unlike more advanced stages with distant metastasis. The overall prevalence of high malnutrition risk in our cohort was 60.2% based on the study criteria. According to the MUST and NRS2002 scores, 9.0% and 52.1% of patients, respectively, met the “high risk” threshold. These findings highlight the substantial variability between nutritional scoring systems, making it difficult to compare results across studies, where PD groups at nutritional risk range from 52% to 83% [23,24,25]. Similarly, although several universal nutritional scoring systems have been developed [26,27,28,29], the limited available evidence prevents definitive conclusions about their comparative accuracy and clinical utility in predicting postoperative morbidity after PD. A retrospective study by La Torre et al. [23] demonstrated that MUST and NRI scores were good predictors of postoperative morbidity and length of hospital stay. However, the only prospective study to date by Probst et al., which analyzed 11 scoring systems in patients undergoing PD, found substantial variability in malnutrition assessment and failed to demonstrate a higher incidence of postoperative complications in these patients [13]. Our findings support these conclusions, indicating that the MUST and NRS2002 scores are unsuitable for predicting postoperative complications risk. However, unlike our study, the patient cohort in Probst et al.’s research did not receive standardized preoperative nutritional interventions for patients at risk. Further research is needed to develop and standardize pancreas-specific nutritional scoring systems.

Analysis of the visceral proteins—albumin and prealbumin—revealed that reduced levels were present not only in the “high-risk” malnutrition cohort but also in patients without restricted food intake and weight loss. Previous research has shown that serum albumin and prealbumin levels are better regarded as inflammatory markers associated with “nutritional risk” rather than as indicators of inadequate nutrient intake [30,31,32], and our results support this view. In our analysis of risk factors for major postoperative complications, the only nutritional parameter with statistical significance was a low preoperative prealbumin level. In contrast to previous studies, the effects of hypoalbuminemia were not observed [33,34]. This result may be influenced by the relatively low number of patients with hypoalbuminemia in our cohort (5.3%) compared with the cohort reported by Uzunoglu et al. (17.0%) [34]. In the absence of baseline laboratory data on prealbumin and albumin levels before nutritional preparation, we could not unequivocally confirm the hypothesis that systemic nutritional support contributed to the normalization of serum levels in some patients before PD.

In our study, the strongest predictor of severe postoperative complications was soft pancreatic texture, followed by pancreatic duct diameter ≤ 3 mm and male sex. These findings are consistent with previously published studies analyzing risk factors for POPF and postoperative morbidity [8,35]. The increased risk of POPF in patients with soft pancreatic tissue is likely related to higher exocrine activity, the frequent presence of a small main pancreatic duct, and the reduced ability of fragile parenchyma to hold sutures without ischemic injury, all of which predispose to anastomotic failure [36]. A nondilated main pancreatic duct increases technical difficulty during anastomosis and reduces the margin for secure suture placement, thereby predisposing to leakage. In addition, nondilated ducts are more often associated with postoperative pancreatic inflammation, further increasing the risk of POPF. A duct diameter below 3 mm is generally regarded as high-risk, and this threshold has been consistently validated in the literature, including by the ISGPS classification that combines duct size and gland texture as predictors of POPF [37]. Given the degree of risk associated with these parameters, PD-related risks may be specific and linked to well-defined factors, thereby limiting the influence of nutritional status on postoperative outcomes.

No significant correlation was found between BMI < 18.5 kg/m^2^ and major postoperative morbidity, possibly due to the small number of patients in this subgroup (four patients). A similarly low proportion of patients undergoing PD with a BMI < 18.5 kg/m^2^ was also reported in a study by Temraz et al. [38], in which no patient met this condition. Unlike the cited study, which demonstrated a significant correlation between weight loss and postoperative morbidity, this relationship was not observed in our study. One possible reason for the differing results may be the influence of preoperative nutritional intervention in our “high-risk” cohort, which included all patients with weight loss.

The main finding of our study is that postoperative morbidity and mortality, as well as the incidence of POPF, PPH, and DGE in the cohort with a “low risk” of malnutrition, are comparable to those in the “high-risk” cohort, whose patients received preoperative nutritional preparation. No comparable prospective observational studies have been reported. A retrospective analysis by Xu et al. [9] demonstrated a lower incidence of POPF in patients with an NRS2002 score ≥ 5 who underwent preoperative nutritional assessment; however, this study did not evaluate overall postoperative morbidity. A possible explanation for our findings—requiring further research—is the effect of preoperative nutritional preparation in high-risk patients, which may enhance nutritional reserves necessary for postoperative healing.

To our knowledge, this is the first published prospective study from Central Europe to focus on nutritional status and risk analysis in patients undergoing PD with systematic preoperative enteral nutritional support. The strength of our study lies in its homogeneous patient group, all of whom underwent a precisely defined PD procedure alongside an unselected range of surgical indications. This constitutes a representative target group for preoperative nutritional screening and intervention.

This study has several limitations. First, it was conducted as a prospective single-center cohort study with a limited number of patients, which may reduce the generalizability of the findings. Within the defined study protocol, nutritional parameters were determined once during preoperative screening and preparation; therefore, a more detailed longitudinal analysis of the effects of nutritional intervention was not feasible. Second, the study design did not include a randomized control group of high-risk patients without nutritional intervention, which limits the ability to draw definitive conclusions on causality between the intervention and outcomes. Third, in patients with chronic pancreatitis, we lacked reliable data on the documented presence or absence of exocrine pancreatic insufficiency. Although all such patients received preoperative pancreatic enzyme substitution, heterogeneity in the degree of exocrine dysfunction cannot be ruled out. Finally, the study did not include a systematic assessment of sarcopenia, which would have required dedicated radiological or functional measurements beyond the scope of the predefined study design. As sarcopenia is increasingly recognized as an important predictor of surgical outcomes, future studies should address this factor explicitly.

## 5. Conclusions

Patients at high risk of malnutrition undergoing PD who received preoperative nutritional support have an incidence of major postoperative complications and POPF comparable to those at low risk of malnutrition. Therefore, preoperative enteral nutritional preparation may be recommended for all patients with weight loss and a lack of appetite associated with restricted oral intake. Low serum prealbumin levels represent a significant nutritional risk factor for major postoperative complications in patients undergoing PD. Neither the MUST nor the NRS2002 scores correlated with the incidence of major postoperative complications of PD. Further research is needed to standardize nutritional assessment and eventually develop pancreas-specific nutritional scoring systems.

## Figures and Tables

**Table 1:** Demographic, pathological and nutritional characteristics of all PD patients included in the study (Olomouc, 2019–2021, *n* = 133) stratified by malnutrition risk level.

Variables	All Patients (*n* = 133, 100.0%)	Malnutrition High Risk (*n* = 80, 60.2%)	Malnutrition Low Risk (*n* = 53, 39.8%)	*p*
**Demographic characteristics**				
Sex				0.655
Male	80 (60.2%)	48 (60.0%)	32 (60.4%)	
Female	53 (39.8%)	32 (40.0%)	21 (39.6%)	
Age (years)	64.2 (36–84)	64.7 (41–84)	64.2 (36–81)	0.862
ASA score				0.771
1	2 (1.5%)	1 (1.3%)	1 (1.9%)	
2	111 (84.1%)	67 (84.8%)	44 (83.0%)	
3	19 (14.4%)	11 (13.9%)	8 (15.1%)	
**Pancreatic characteristics**				
Pancreatic texture				0.578
Soft	37 (27.8%)	20 (25.0%)	17 (32.1%)	
medium/hard	96 (72.2%)	60 (75.0%)	36 (67.9%)	
Pancreatic duct diameter				0.712
≤3 mm	52 (39.4%)	30 (38.0%)	22 (41.5%)	
4 mm	43 (32.6%)	26 (32.9%)	17 (32.1%)	
≥5 mm	37 (28.0%)	23 (29.1%)	14 (26.4%)	
Pathology				0.636
PDAC	74 (55.6%)	42 (52.5%)	32 (60.4%)	
Ampullary carcinoma	14 (10.5%)	9 (11.3%)	5 (9.4%)	
Cholangiocarcinoma	24 (18.0%)	17 (21.3%)	7 (13.2%)	
Chronic pancreatitis	11 (8.3%)	5 (6.3%)	6 (11.3%)	
Duodenal carcinoma	5 (3.8%)	5 (6.3%)	0 (0.0%)	
Other	5 (3.8%)	2 (2.5%)	3 (5.7%)	
**Laboratory results**				
Albumin < 35 g/L	7 (5.3%)	4 (5.0%)	3 (5.7%)	0.796
Prealbumin < 0.2 g/L	14 (10.5%)	10 (12.5%)	4 (7.5%)	0.586
**Nutritional characteristics**				
BMI (kg/m^2^)	25.9 (17.5–38.6)	25.8 (17.5–38.6)	26.9 (21.1–36.3)	0.356
Lack of appetite	12 (9.0%)	12 (15.0%)	0 (0.0%)	
Weight loss				
without Weight loss	63 (47.4%)	10 (12.5%)	53 (100.0%)	
>0 kg ≤5 kg	20 (15.0%)	20 (25.0%)	0 (0.0%)	
>5 kg ≤10 kg	30 (22.6%)	30 (37.5%)	0 (0.0%)	
>10 kg	20 (15.0%)	20 (25.0%)	0 (0.0%)	
≤5% body weight	75 (56.4%)	22 (27.5%)	53 (100.0%)	
>5% ≤10% body weight	24 (18.0%)	24 (30.0%)	0 (0.0%)	
>10% body weight	34 (25.6%)	34 (42.5%)	0 (0.0%)	
MUST score ≥ 2	12 (9.0%)	12 (15.0%)	0 (0.0%)	
NRS2002 score ≥ 3	72 (54.1%)	72 (90.0%)	0 (0.0%)	

Qualitative data were expressed as *n* (%) and quantitative data as median (min–max); ASA, American Society of Anesthesiologists; BMI, Body mass index; MUST, malnutrition universal screening Tool; NRS2002, Nutritional Risk Screening Score 2002; PDAC, Pancreatic ductal adenocarcinoma.

**Table 2:** Operative and postoperative characteristics of all patients with PD included in the study (Olomouc, 2019–2021, *n* = 133) stratified by malnutrition risk.

Variables	All Patients (*n* = 133, 100.0%)	Malnutrition High Risk (*n* = 80, 60.2%)	Malnutrition Low Risk (*n* = 53, 39.8%)	*p*
Type of resection				0.638
Pylorus-preserving	121 (91.0%)	75 (93.8%)	46 (86.8%)	
Whipple	12 (9.0%)	5 (6.3%)	7 (13.2%)	
Operative time (min)	300 (198–519)	300 (198–421)	300 (217–519)	0.782
Blood loss (mL)	425 (100–5800)	400 (100–1800)	500 (100–5800)	0.341
Time to surgery (days)	21(5–42)	22 (10–42)	21(5–38)	0.498
Hospital stay (days)	14 (7–128)	14 (7–128)	14 (7–75)	0.668
Mortality				
30-day	4 (3.0%)	3 (3.8%)	1 (1.9%)	0.809
90-day	4 (3.0%)	3 (3.8%)	1 (1.9%)	0.809
POPF				0.777
B	16 (12.0%)	9 (11.3%)	7 (13.2%)	
C	7 (5.3%)	6 (7.5%)	1 (1.9%)	
DGE				0.822
A	6 (4.5%)	4 (5.1%)	2 (3.8%)	
B	21(15.8%)	10 (12.5%)	11 (20.8%)	
C	7 (5.3%)	7 (8.8%)	0 (0.0%)	
PPH				0.675
A	0 (0.0%)	0 (0.0%)	0 (0.0%)	
B	7 (5.3%)	5 (6.3%)	2 (3.8%)	
C	5 (3.8%)	2 (2.5%)	3 (5.7%)	
Clavien-Dindo grade				0.721
0	32 (24.1%)	19 (23.8%)	13 (24.5%)	
I	19 (14.3%)	13 (16.3%)	6 (11.3%)	
II	54 (40.6%)	31 (38.8%)	23 (43.4%)	
IIIa	2 (1.5%)	1 (1.3%)	1 (1.9%)	
IIIb	14 (10.5%)	10 (12.5%)	4 (7.5%)	
IVa	5 (3.8%)	2 (2.5%)	3 (5.7%)	
IVb	3 (2.3%)	1 (1.3%)	2 (3.8%)	

Qualitative data are expressed as *n* (%) and quantitative data as median (min-max); DGE, delayed gastric emptying; POPF, postoperative pancreatic fistula; PPH, postoperative pancreatic hemorrhage.

**Table 3:** Univariate and multivariate analysis of risk factors for major postoperative morbidity of PD patients (Olomouc, 2019–2021, *n* = 133).

		Univariate Analysis		Multivariate Analysis	
Variables	Category	OR (95% CI)	*p*	OR (95% CI)	*p*
Sex	Male/Female	**2.677 (1.310–5.470)**	**0.007**	**2.240 (1.096–4.578)**	**0.027**
Age	≥70 vs. <70	0.838 (0.405–1.734)	0.634		
ASA score	III vs. I/II	0.871 (0.522–1.453)	0.597		
BMI	<18.5 vs. ≥18.5	0.820 (0.213–3.157)	0.773		
Lack of appetite	Yes/No	0.863 (0.357–2.086)	0.744		
Weight loss	>5 kg vs. ≤5 kg	0.935 (0.542–1.613)	0.809		
	>0% vs. ≤10% body weight	1.298 (0.668–2.522)	0.442		
Malignancy	Yes/No	0.784 (0.281–2.187)	0.642		
Pancreatic duct diameter	≤3 mm vs. >3 mm	**3.640 (1.955–6.777)**	**<0.001**	**3.507 (1.336–9.206)**	**0.011**
Pancreatic texture	Soft vs. medium/hard	**5.416 (2.116–13.863)**	**<0.001**	**4.331 (1.828–10.261)**	**<0.001**
Albumin (g/L)	<35 vs. ≥35	0.739 (0.335–1.630)	0.454		
Prealbumin (g/L)	<0.2 vs. ≥0.2	**3.562 (1.279–9.920)**	**0.015**	**3.815 (1.081–13.464)**	**0.037**
MUST	≥2 vs. ≤1	0.913 (0.362–2.303)	0.847		
NRS2002	≥3 vs. ≤2	0.861 (0.415–1.786)	0.688		

Differences in major morbidity were analyzed using a multivariable logistic regression model; Values in bold are statistically significant; ASA, American Society of Anesthesiologists; BMI, Body mass index; CI, Confidence interval; MUST, Malnutrition Universal Screening Tool; NRS2002, Nutritional Risk Screening Score 2002; OR, Odds Ratio.

## Data Availability

The original data presented in the study are openly available at https://upload.upol.cz/E8B1-6NUZ (accessed on 6 August 2025).

## Abbreviations

The following abbreviations are used in this manuscript:

ASA	American Society of Anesthesiologists
BMI	Body mass index
CD	Clavien-Dindo classification
CI	Confidence interval
CT	Computed tomography
DGE	Delayed gastric emptying
ESPEN	European Society of Nutrition and Metabolism
MUST	Malnutrition Universal Screening Tool
NRS2002	Nutritional Risk Screening Score 2002
NSQIP	Surgical Quality Improvement Program
OR	Odds Ratio
PD	Pancreaticoduodenectomy
PDAC	Pancreatic ductal adenocarcinoma
POPF	Postoperative pancreatic fistula
PPH	Postoperative pancreatic hemorrhage
WHO	World Health Organization

## References

[1] Gianotti L., Besselink M.G., Sandini M., Hackert T., Conlon K., Gerritsen A., Griffin O., Fingerhut A., Probst P., Hilal A. (2018). Nutritional support and therapy in pancreatic surgery: A position paper of the International Study Group on Pancreatic Surgery (ISGPS). Surgery.

[2] Witzigmann H., Diener M.K., Kienkötter S., Rossion I., Bruckner T., Werner B., Humar B., Welsch T., Croner R., Schlitt H.J. (2016). No need for routine drainage after pancreatic head resection: The dual-center, randomized, controlled PANDRA trial (ISRCTN04937707). Ann. Surg..

[3] Keck T., Wellner U.F., Bahra M., Klein F., Sick O., Niedergethmann M., Wilhelm T.J., Farkas S., Börner T., Bruns C. (2016). Pancreatogastrostomy versus pancreatojejunostomy for RECOnstruction After PANCreatoduodenectomy (REC0-PANC, DRKS 00000767): Perioperative and long-term results of a multicenter randomized controlled trial. Ann. Surg..

[4] Halle-Smith J.M., Powell-Brett S.F., Hall L.A., Duggan S.N., Griffin O., Phillips M.E., Roberts K.J. (2023). Recent advances in pancreatic ductal adenocarcinoma: Strategies to optimize perioperative nutritional status in pancreatoduodenectomy patients. Cancer.

[5] Bassi C., Marchegiani G., Dervenis C., Sarr M., Abu Hilal M., Adham M., Allen P., Andersson R., Asbun H.J., Besselink M.G. (2017). International Study Group on Pancreatic Surgery (ISGPS). The 2016 update of the International Study Group (ISGPS) definition and grading of postoperative pancreatic fistulas 11 years after. Surgery.

[6] Wente M.N., Veit J.A., Bassi C., Dervenis C., Fingerhut A., Gouma D.J., Izbicki J.R., Neoptolemos J.P., Padbury R.T., Sarr M.G. (2007). Postpancreatectomy hemorrhage (PPH): International Study Group for Pancreatic Surgery (ISGPS) definition. Surgery.

[7] Wente M.N., Bassi C., Dervenis C., Fingerhut A., Gouma D.J., Izbicki J.R., Neoptolemos J.P., Padbury R.T., Sarr M.G., Traverso L.W. (2007). Delayed gastric emptying (DGE) after pancreatic surgery: A definition suggested by the International Study Group of Pancreatic Surgery (ISGPS). Surgery.

[8] Mungroop T.H., Klompmaker S., Wellner U.F., Steyerberg E.W., Coratti A., D’Hondt M., de Pastena M., Dokmak S., Khatkov I., Saint-Marc O. (2021). European Consortium on Minimally Invasive Pancreatic Surgery (E-MIPS). Updated Alternative Fistula Risk Score (ua-FRS) to Include Minimally Invasive Pancreatoduodenectomy: Pan-European Validation. Ann. Surg..

[9] Xu J.Y., Tian X.D., Song J.H., Chen J., Yang Y.M., Wei J.M. (2021). Preoperative nutritional support may reduce the prevalence of postoperative pancreatic fistulas after open pancreaticoduodenectomy in patients with high nutritional risk as determined by the NRS2002. BioMed Res. Int..

[10] Loveček M., Záruba P., Ulrych J., Froněk J., Oliverius M., Čečka F., Hlavsa J., Šimša J., Sirotek L., Hladík P. (2023). Minimally-invasive pancreatic surgery in high volume centers in the Czech Republic—Current status and possible implementations. Rozhl. Chir..

[11] Weimann A., Braga M., Carli F., Higashiguchi T., Hübner M., Klek S., Laviano A., Ljungqvist O., Lobo D.N., Martindale R. (2017). ESPEN guideline: Clinical nutrition in surgery. Clin. Nutr..

[12] Takagi K., Domagala P., Hartog H., van Eijck C., Groot Koerkamp B. (2019). Current evidence of nutritional therapy in pancreatoduodenectomy: Systematic review of randomized controlled trials. Ann. Gastroenterol. Surg..

[13] Probst P., Haller S., Bruckner T., Ulrich A., Strobel O., Hackert T., Diener M.K., Büchler M.W., Knebel P. (2017). Prospective trial to evaluate the prognostic value of different nutritional assessment scores in pancreatic surgery (NURIMAS Pancreas). Br. J. Surg..

[14] Dindo D., Demartines N., Clavien P.A. (2004). Classification of surgical complications: A new proposal with evaluation in a cohort of 6336 patients and survey results. Ann. Surg..

[15] World Health Organisation (2000). Obesity: Prevention and Management of a Global Epidemic. Report of the WHO Consultation.

[16] Mękal D., Sobocki J., Badowska-Kozakiewicz A., Sygit K., Cipora E., Bandurska E., Czerw A., Deptała A. (2023). Evaluation of nutritional status and the impact of nutritional treatment in patients with pancreatic cancer. Cancers.

[17] Vaid S., Bell T., Grim R., Ahuja V. (2012). Predicting risk of death in general surgery patients on the basis of preoperative variables using American College of Surgeons National Surgical Quality Improvement Program data. Perm. J..

[18] Ausania F., Senra P., Meléndez R., Caballeiro R., Ouviña R., Casal-Núñez E. (2019). Prehabilitation in patients undergoing pancreaticoduodenectomy: A randomized controlled trial. Rev. Esp. Enferm. Dig..

[19] Bundred J.R., Kamarajah S.K., Hammond J.S., Wilson C.H., Prentis J., Pandanaboyana S. (2020). Prehabilitation prior to surgery for pancreatic cancer: A systematic review. Pancreatology.

[20] van der Gaag N.A., Rauws E.A., van Eijck C.H., Bruno M.J., van der Harst E., Kubben F.J., Gerritsen J.J., Greve J.W., Gerhards M.F., de Hingh I.H.J.T. (2010). Preoperative biliary drainage in pancreatic head cancer. N. Engl. J. Med..

[21] Karagianni V.T., Papalois A.E., Triantafillidis J.K. (2012). Nutritional status and support before and after pancreatectomy for patients with pancreatic cancer and chronic pancreatitis. Indian J. Surg. Oncol..

[22] Kempeneers M.A., Issa Y., Ahmed Ali U., Baron R.D., Besselink M.G., Büchler M.W., Erkan M., Fernandez-Del Castillo C., Isaji S., Izbicki J.R. (2020). Working group for the International (IAP—APA—JPS—EPC) Consensus Guidelines for Chronic Pancreatitis. International consensus guidelines for surgery and timing of intervention in chronic pancreatitis. Pancreatology.

[23] La Torre M., Ziparo V., Nigri G., Cavallini M., Balducci G., Ramacciato G. (2013). Malnutrition and pancreatic surgery: Prevalence and outcomes. J. Surg. Oncol..

[24] Kim E., Kang J.S., Han Y., Kim H., Kwon W., Kim J.R., Kim S.W., Jang J.Y. (2018). Influence of preoperative nutritional status on clinical outcomes after pancreatoduodenectomy. HPB.

[25] Kang J., Park J.S., Yoon D.S., Kim W.J., Chung H., Lee S.M., Chang N. (2016). Dietary intake and nutritional status of Pancreatic Cancer Surgical Patients. Clin. Nutr. Res..

[26] Kondrup J., Allison S.P., Elia M., Vellas B., Plauth M., Educational and Clinical Practice Committee, European Society of Parenteral and Enteral Nutrition (ESPEN) (2003). ESPEN Guidelines for Nutrition Screening, 2002. Clin. Nutr..

[27] British Association for Parenteral and Enteral Nutrition (2003). The UK ‘MUST’ Explanatory Booklet. A Guide to the ‘Malnutrition Universal Screening Tool’ (‘MUST’) for Adults. http://www.bapen.org.uk/pdfs/must/must_explan.pdf.

[28] Detsky A., McLaughlin J.R., Baker J., Johnston N., Whittaker S., Mendelson R., Jeejeebhoy K.N. (1987). What is a subjective global assessment of nutritional status?. J. Parenter. Enteral. Nutr..

[29] Rubenstein L.Z., Harker J.O., Salvà A., Guigoz Y., Vellas B. (2001). Screening for undernutrition in geriatric practice: Developing the shortform mini-nutritional assessment (MNASF). J. Gerontol. A Biol. Sci. Med. Sci..

[30] Yeh D.D., Johnson E., Harrison T., Kaafarani H.M.A., Lee J., Fagenholz P., Saillant N., Chang Y., Velmahos G. (2018). Serum albumin and prealbumin levels did not correlate with nutrient delivery in surgical intensive care unit patients. Nutr. Clin. Pract..

[31] Lee J.L., Oh E.S., Lee R.L., Finucane T.E. (2015). Serum albumin and prealbumin in calorically restricted, non-diseased individuals: A systematic review. Am. J. Med..

[32] Evans D.C., Corkins M.R., Malone A., Miller S., Mogensen K.M., Guenter P., Jensen G.L., ASPEN Malnutrition Committee (2021). Use of visceral proteins as nutrition markers: An ASPEN Position Paper. Nutr. Clin. Pract..

[33] Bozzetti F., Gianotti L., Braga M., Di Carlo V., Mariani L. (2007). Postoperative complications in patients with gastrointestinal cancer: The joint role of nutritional status and support. Clin. Nutr..

[34] Uzunoglu F.G., Reeh M., Vettorazzi E., Ruschke T., Hannah P., Nentwich M.F., Vashist Y.K., Bogoevski D., König A., Janot M. (2014). Preoperative pancreatic resection (PREPARE) score: A prospective multicenter-based morbidity risk score. Ann. Surg..

[35] Akgul O., Merath K., Mehta R., Hyer J.M., Chakedis J., Wiemann B., Johnson M., Paredes A., Dillhoff M., Cloyd J. (2019). Postoperative pancreatic fistula after pancreaticoduodenectomy: Stratification of patient risk. J. Gastrointest. Surg..

[36] Eshmuminov D., Schneider M.A., Tschuor C., Raptis D.A., Kambakamba P., Müller X., Kuemmerli C., Shah K., Limani P., Lesurtel M. (2018). Systematic review and meta-analysis of postoperative pancreatic fistula rates using the updated 2016 International Study Group Pancreatic Fistula definition in patients undergoing pancreatic resection with soft and hard pancreatic texture. HPB.

[37] Schuh F., Mihaljevic A.L., Probst P., Trudeau M.T., Müller P.C., Marchegiani G., Besselink M.G., Uzunoglu F., Izbicki J.R., Falconi M. (2023). A simple classification of pancreatic duct size and texture predicts postoperative pancreatic fistula: A classification of the International Study Group of Pancreatic Surgery. Ann. Surg..

[38] Temraz S., Charafeddine M., Khalifeh M.J., Shamseddine A. (2025). Preoperative markers of postoperative complications in pancreatic cancer patients: A single-center study. J. Epidemiol. Glob. Health.

